# Avascular Necrosis of Both Hips From Iatrogenic Cushing ’s Syndrome due to Coadministration of Fluticasone and Ritonavir in an HIV-Infected Patient

**DOI:** 10.7759/cureus.9644

**Published:** 2020-08-10

**Authors:** Ravi Kant, Mark D Cromer, Rashmi Chandra, Kashif Munir, Vipin Verma

**Affiliations:** 1 Endocrinology, Diabetes and Metabolism, Medical University of South Carolina, Anderson, USA; 2 Internal Medicine, University of Alabama at Birmingham, Birmingham, USA; 3 Internal Medicine, Medical University of South Carolina, Anderson, USA; 4 Endocrinology, Diabetes and Metabolism, University of Maryland Medical Center, Baltimore, USA; 5 Geriatrics, Medical University of South Carolina, Anderson, USA

**Keywords:** general medicine pharmacology, iatrogenic cushing’s syndrome, fluticasone, hiv, protease inhibitors, ritonavir, avascular osteonecrosis, inhaled corticosteroids, avascular necrosis (avn), drug-drug interactions

## Abstract

We report a case of avascular necrosis (AVN), hypercalcemia, and iatrogenic Cushing’s syndrome in an HIV-positive patient taking inhaled (ICS) and nasal corticosteroids fluticasone and ritonavir.

A 45-year-old HIV-infected African-American woman was seen for initial evaluation for multinodular goiter in December 2015. Relevant medications were ritonavir, raltegravir, darunavir, fluticasone propionate HFA, and nasal fluticasone propionate. Physical examination revealed classical cushingoid appearance but laboratory testing showed abnormal adrenocorticotropic hormone (ACTH) stimulation test. A diagnosis of iatrogenic Cushing’s syndrome due to inhibition of fluticasone metabolism from protease inhibitor (PI) therapy with secondary adrenal suppression was made. Fluticasone nasal spray and HFA were discontinued and hydrocortisone replacement dose was initiated. The patient’s Cushing’s related symptoms improved over several months. Follow-up evaluation showed non-parathyroid hormone-mediated hypercalcemia. A detailed laboratory evaluation looking for the etiology for hypercalcemia was unremarkable except for an elevated urine N-telopeptide/creatinine ratio. Meanwhile, the patient developed a new symptom of hip pain. MRI of both hips showed bilateral AVN. Sickle cell screen was negative and a right hip replacement was completed in May 2017.

Since this is the fourth case report of AVN from iatrogenic Cushing’s syndrome in an HIV-infected patient taking a PI and ICS concomitantly, there is more likely a causal relationship and not simply a coincidental finding. Extreme caution should be used when considering any ICS therapy in combination with PIs in HIV-infected patients.

## Introduction

Highly active antiretroviral therapy (HAART) is the mainstay treatment for patients infected with human immunodeficiency virus (HIV). Ritonavir is a protease inhibitor (PI) that blocks a cleavage step necessary for the replication of HIV and is typically given in combination with other HIV medications to boost their efficacy by acting as an inhibitor of hepatic cytochrome P450 enzyme 3A4 (CYP3A4). However, coadministration of ritonavir with any compound that is metabolized via CYP3A4 enzymes will inevitably lead to elevated plasma concentrations of that compound, prolonging its action and causing potential adverse systemic effects. Inhaled (ICS) and nasal corticosteroids, such as fluticasone, are known substrates of CYP3A4. Thus, it stands to reason that coadministration of ritonavir and fluticasone can induce adverse effects of prolonged, elevated glucocorticoids, such as Cushing’s syndrome, osteoporosis, or avascular necrosis (AVN), and tertiary adrenal insufficiency after withdrawal of glucocorticoids [1-5]. We report a case of AVN from iatrogenic Cushing’s syndrome in an HIV-positive patient taking inhaled and nasal fluticasone and ritonavir.

## Case presentation

A 45-year-old HIV-infected African-American woman was seen for initial evaluation for multinodular goiter in December 2015. The patient presented with a cushingoid appearance and classic findings of wide purple striae, central obesity, round facies, and proximal muscle weakness. She was a poor historian on the initial visit and was unable to provide an adequate account of the progression of her cushingoid symptoms. Other notable medical history included HIV infection, asthma, hypertension, and obstructive sleep apnea. Relevant medications were ritonavir 10 mg PO twice daily, raltegravir 400 mg PO twice daily, darunavir 800 mg PO once daily, fluticasone propionate HFA (110 mcg/ACT Inhalation Aerosol) one puff twice daily, and fluticasone propionate (50 mcg/ACT Nasal Suspension) two sprays in each nostril once daily.

**Figure 1:**
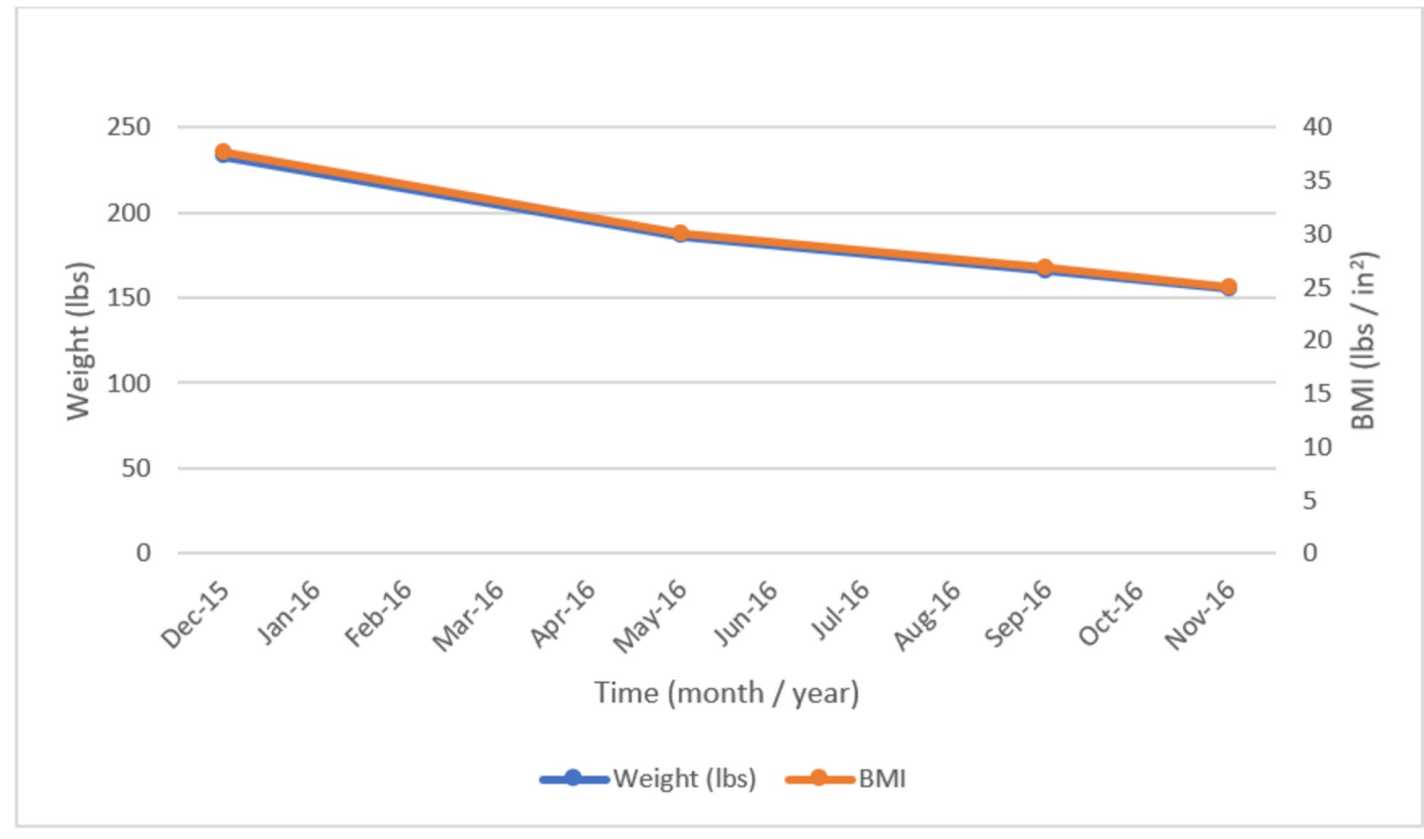
Body mass index (BMI) and weight changes after stopping inhaled and nasal fluticasone.

Physical examination at the initial evaluation showed an obese female with a body mass index (BMI) of 37.6 (Figure 1); she had “moon-like facies”, significant dorsocervical and supraclavicular fat pads, and wide purple striae on the abdomen. The patient’s clinical picture was consistent with Cushing’s syndrome. However, a 1 mg dexamethasone suppression test (DST) was normal and 24-hour urine cortisol was undetectable, raising suspicion for adrenal insufficiency. A follow-up ACTH stimulation test was also abnormal with baseline cortisol of 0 pmol/L and 60 minutes stimulated cortisol level of 9 pmol/L and low normal ACTH of 9.1 pg/ml (7.2-63.3) confirming the diagnosis of adrenal insufficiency (Table 1). The patient was then initiated on hydrocortisone 10 mg PO twice daily.

**Table 1:** Laboratory evaluation for Cushing’s syndrome and hypercalcemia. DST: dexamethasone suppression test, ACTH: adrenocorticotropic hormone; PTH: parathyroid hormone, NTX: N-terminal telopeptide, BCE: bone collagen equivalent, PTH-rP: parathyroid hormone-related protein, SPEP: serum protein electrophoresis, UPEP: urine protein electrophoresis.

Laboratory test (normal reference range)		December 2015	April 2016	September 2016	November 2016	December 2016	August 2017
1 mg DST– am cortisol (<1.8 mcg/dl)		1 (normal)					
24-hr urinary free cortisol (0-50 mcg/24 hr)		Undetectable					
ACTH stimulation test (>18 mcg/dl)	Result	Abnormal	Abnormal	Normal	Normal		
	0-min cortisol	0	2	10	10		
	30-min cortisol	6	8	22	25		
	60-min cortisol	9	11	22	24		
ACTH (7.2-63.3 pg/ml)		9.1	9.1				
Serum calcium (8.6-10.0 mg/dl)				11.0	10.5	10.2	9.2
Albumin (g/dl)				4.0	3.9	3.9	4.0
PTH (15-65 pg/ml)				11.0		11.1	23.9
Serum 25 OH vitamin D (30-100 ng/ml)				24	24		27
Serum vitamin D 1, 25 dihydroxy (19.9-79.3 pg/ml)				<5	< 5		
Phosphorus (2.5-4.5 mg/dl)				3.1			4.0
NTX/creatinine (0-89 nM BCE/nM Cr)				139			
PTH-rP (pmol/L)				Undetectable			
SPEP and UPEP				No monoclonal bands			
Serum vitamin A level					Normal		

It was discovered that the patient had been taking HAART since 1996 in addition to fluticasone nasal spray for several years. A diagnosis of iatrogenic Cushing’s syndrome due to inhibition of fluticasone metabolism from PI therapy with secondary adrenal suppression was made. Fluticasone nasal spray and HFA were discontinued, and hydrocortisone dose was increased to the maintenance dose.

**Figure 2:**
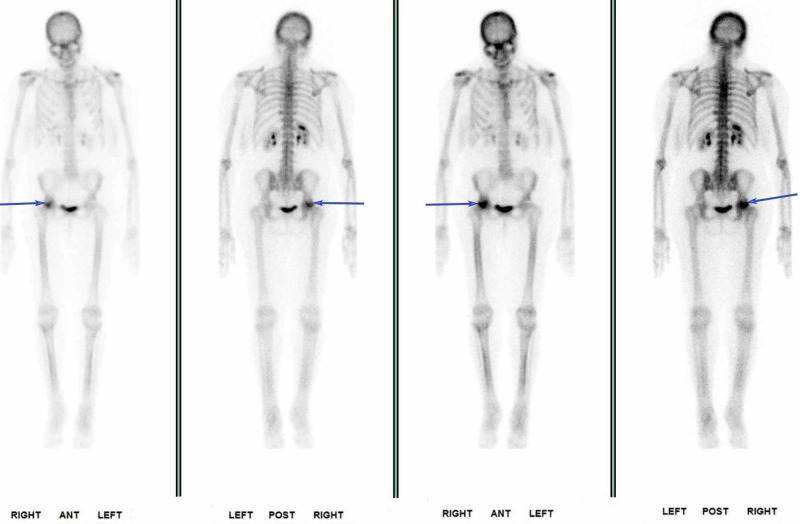
Nuclear medicine bone scan showing a focal increase in radiotracer uptake within the right proximal femur.

Cushing’s related symptoms improved over several months after stopping fluticasone with the resolution of cushingoid facies. Weight loss of 80 lbs was achieved by September 2016. An ACTH stimulation test was normal in September 2016, indicating recovery of adrenal function (Table 1). Patient self-discontinued hydrocortisone at that time. Interestingly, the patient developed non-PTH-mediated hypercalcemia. Adrenal insufficiency was excluded by normal ACTH stimulation testing. A detailed lab evaluation looking for etiology for hypercalcemia was unremarkable except for elevated urine N-telopeptide/creatinine ratio of 139 nM bone collagen equivalents (BCE)/nM Cr (0-89). In November 2016, an ACTH stimulation test was again normal and the patient’s weight had begun to stabilize (Figure 1). X-rays of the chest and hip did not show any pathology related to hypercalcemia. However, a dual-energy x-ray absorptiometry (DEXA) scan demonstrated osteopenia. While the etiology of hypercalcemia remained unclear, new symptoms of hip pain developed. A nuclear medicine three-phase bone scan demonstrated non-specific focally increased uptake within the right proximal femur (Figure 2). 

**Figure 3:**
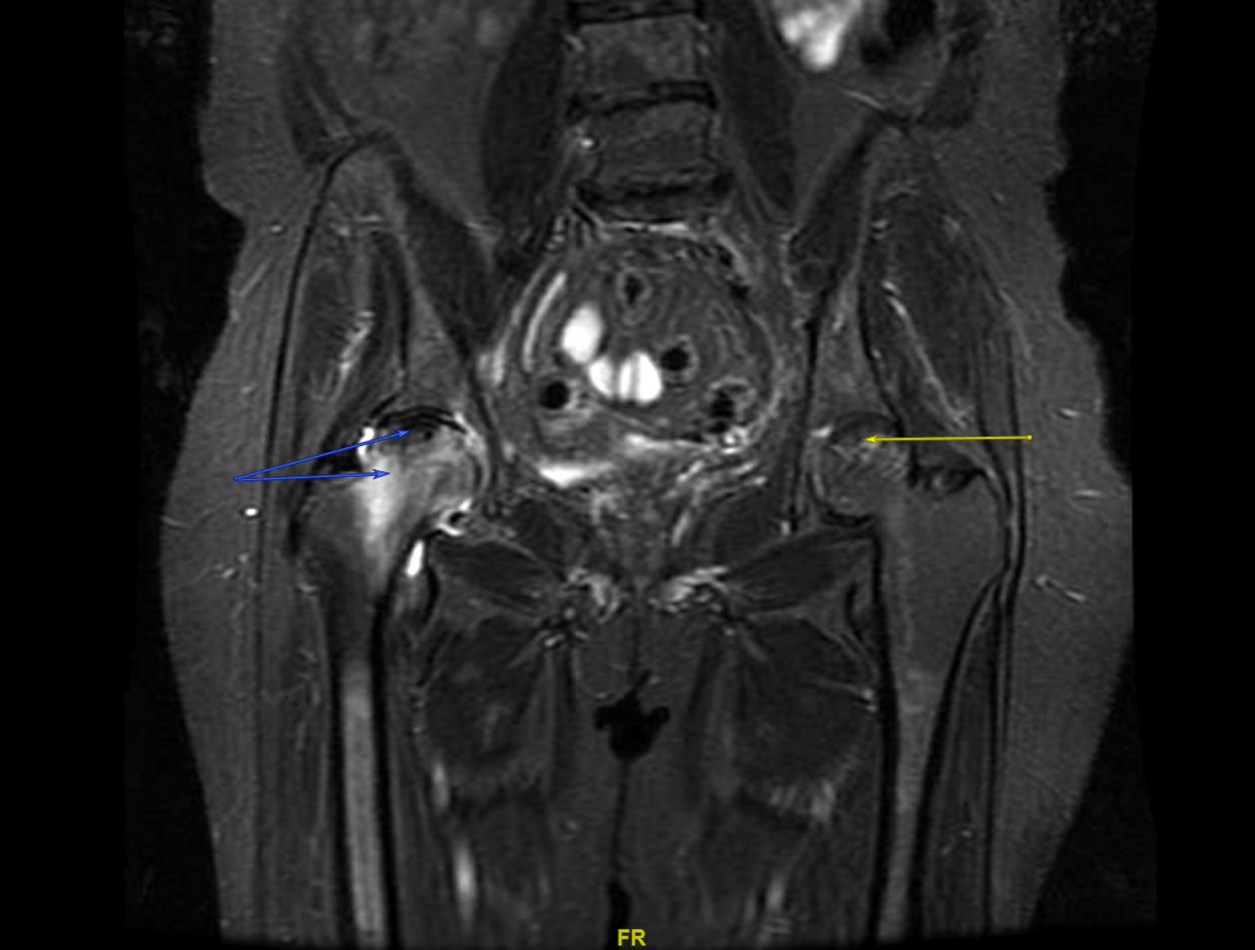
MRI of the pelvis and both hips without contrast showing subacute avascular necrosis of the left hip (yellow arrow), and acute, subacute, and chronic avascular necrosis of the right hip (blue arrows).

MRIs of both hips showed bilateral AVN of the right hip with acute, subacute, and chronic findings, and subacute changes of the left hip (Figure 3). Sickle cell screen was negative and a right hip replacement was completed in May 2017. Since the patient developed non-PTH mediated hypercalcemia after adrenal functions returned to normal, hypercalcemia was deemed secondary to AVN. Serum calcium normalized over the next few months (Table 1). At follow-up in August 2017, serum calcium levels remained normal and both Cushing's syndrome and adrenal insufficiency had resolved. 

## Discussion

Our patient presented with clinical cushingoid features in the setting of biochemical adrenal insufficiency consistent with the diagnosis of iatrogenic Cushing’s syndrome leading to secondary adrenal suppression. Iatrogenic Cushing’s syndrome due to inhibition of hepatic CYP3A4 metabolism of inhaled and nasal fluticasone by PI is well documented in the literature [1-5]. However, our case presented with non-PTH-mediated hypercalcemia and AVN of bilateral hips, most likely complications of iatrogenic Cushing’s syndrome. HIV-infected patients have been reported to be at high risk for AVN with one study showing approximately 100-fold greater risk compared to the general population [6]. The risk of AVN is particularly higher in patients on PI therapy; however, the mechanism is unclear [7]. A detailed search of the literature only resulted in three other cases of AVN presenting as a complication of coadministering ritonavir and fluticasone [8-10]. Kaviani et al. reported a case of a 60-year-old man with AVN of bilateral hips in an HIV-positive patient taking ICS with ritonavir-boosted antiretroviral therapy [8]. Similarly, a case of bilateral knee AVN in an HIV-infected man receiving inhaled fluticasone and PI was reported by Pollett et al. [9].

Drug-drug interactions are one of the most common causes of adverse drug reactions (ADRs). Polypharmacy, as was present in this patient’s case, leads to the greater complexity of therapeutic management, which inevitably leads to a higher risk of drug interactions that can induce ADRs by either increasing or decreasing the efficacy of medications [11]. PIs are known inhibitors of the CYP3A4 liver enzymes and act as pharmacokinetic enhancers for other compounds metabolized by this enzyme [12]. Fluticasone is an ICS that is also metabolized by the CYP3A4 enzymes. It has the highest suppressive effect compared to other inhaled steroids on the hypothalamus-pituitary-adrenal (HPA) axis with higher lipophilicity, longer elimination half-life, a higher volume of distribution, and prolonged glucocorticoid relative receptor affinity [13]. Other types of ICSs such as beclomethasone or budesonide may be safer due to their lower receptor binding affinity and shorter half-life; however, they are also metabolized via CYP3A4 and cases of Cushing’s syndrome have been documented with their use as well [14]. Thus, it stands to reason that an individual taking both a PI and an ICS such as fluticasone could cause prolonged periods of elevated serum glucocorticoid concentration and induce an iatrogenic Cushing’s syndrome with its associated complications. Furthermore, in our case, stopping fluticasone resulted in adrenal insufficiency due to prolonged suppression of the HPA axis from elevated serum fluticasone level. Our patient’s condition significantly improved after stopping fluticasone and initiating an oral corticosteroid in its place.

AVN is the destruction of bone tissue due to disrupted blood flow. AVN is divided into two main categories: traumatic and non-traumatic. While traumatic is the most common cause of AVN, the two most common causes of non-traumatic AVN are due to exogenous glucocorticoids and alcohol. Usually, AVN results from chronic administration of high-dose steroids, but there have been reports of AVN in patients using ICS [15]. Thus, AVN could be a severe complication of coadministering ritonavir and fluticasone as presented in our case. Since this is the fourth case report of AVN from iatrogenic Cushing’s syndrome in an HIV-infected patient taking a PI and ICS concomitantly, there is more likely a causal relationship and not simply a coincidental finding. Our case provides a possible explanation for the high incidence of AVN in HIV-infected patients who are on PIs.

## Conclusions

Our case underscores the importance of carefully reviewing medications in HIV-infected patients, who usually get prescriptions from multiple providers. Even though iatrogenic Cushing’s syndrome from coadministration of a PI and ICS is well documented in the literature, we report only the fourth case of AVN in an HIV-infected patient taking ritonavir and inhaled and nasal fluticasone. AVN should be considered in the differential diagnosis for joint pain in HIV-positive patients, particularly if they are prescribed PI and ICS. When treating HIV patients with HAART, potential pharmacological interactions due to altered cytochrome P450 activity need to be considered. If Cushing’s syndrome is suspected, rapid cessation of the offending medication will help prevent unnecessary risks of patient morbidity and mortality. Thus, extreme caution should be used when considering any ICS therapy in combination with HAART involving PIs such as ritonavir.
